# Image dataset of benthic foraminifera taxa from Denmark Strait sediments on the northwestern Iceland slope (North Atlantic Ocean)

**DOI:** 10.1016/j.dib.2022.108040

**Published:** 2022-03-11

**Authors:** Liubov Kireenko, Anna Tikhonova, Nina Kozina, Alexander Matul

**Affiliations:** Shirshov Institute of Oceanology, Russian Academy of Sciences, Russia

**Keywords:** Benthic foraminifera, Denmark Strait, North Atlantic Ocean, Micropaleontology, Microphotograph

## Abstract

The article presents an image dataset of benthic foraminifera assemblage in long sediment core AMK-5890 obtained during the 71st cruise of the research vessel Akademic Mstislav Keldysh in summer 2018 from the northwestern Iceland slope in Denmark Strait (North Atlantic Ocean). In the area of study, dense cold water masses of the East Greenland Current from the Nordic Seas contact and mix with warm salty water of the Irminger Current from the North Atlantic Ocean creating specific conditions for the benthic microfauna habitat which reflect forming the lower link of the global thermohaline circulation. The benthic foraminifera assemblages are a reliable indicator for paleooceanographic and ecological reconstructions describing the environments on the bottom in present and past. In the core AMK-5890, the benthic foraminifera assemblage consists of 63 species some of which were combined into one genus (for example, *Fissurina* sp.). The dominants, subdominants and species list of benthic foraminifera changed within the core depth. This image dataset helps to fill the gaps in the practical micropaleontological analysis of benthic foraminifera giving better information for the species identification from the modern and Pleistocene sediments.

## Specifications Table

**Table:** 

Subject	Palaeontology
Specific subject area	Benthic foraminifera
	Sediment core
Type of data	Tables
	Figure
	Phototable
How the data were acquired	Samples were obtained during expedition in North Atlantic Ocean in the summer 2018; core was sampled by large-diameter pipe (LDP); benthic foraminifera were identified to the species level and counted under the Nikon microscope SMZ800N; photos were made using a Nikon microscope SMZ25 equipped with a Nikon camera DS-Fi3 and NIS-Elements D software. Microphotograph tables were made in the computer softwares Adobe Photoshop CC 2019 and CorelDRAW 2020.
Data format	Raw
Description of data collection	The samples were identified under the stereomicroscope, benthic foraminifera tests were counted and selected for the microphotography according to the typical description of species
Data source location	Core AMK-5890 (65°45.081′ s.w.; 25°37.822′ s.d.) was selected in the 71st cruise of the R/V "Akademik Mstislav Keldysh" in summer 2018. The core depth is 268 m.
Data accessibility	The data are available with this article

**Value of the Data**

• This dataset presents high-quality images of benthic foraminifera species in core from Denmark Strait, climatically sensitive area of North Atlantic Ocean located at the junction of the boreal and Arctic regions.
• It can be useful in palaeoceanographic and paleoenvironmental studies of Denmark Strait and Iceland shelf.
• The data are reused for further studies of recoveries palaeoceanographic conditions of past and Quaternary stratigraphy.
• It can help fill in the gaps in images of benthic foraminifera species in the high northern latitudes of the Atlantic Ocean and it is of great methodological importance for micropaleontological studies of Meso-Cenozoic associations of foraminifera.
• Image dataset are valuable for micropaleontologists and biologists in better routine species identification of benthic foraminifera in North Atlantic sediments.

## 1 Data Description

**Fig. 1:**
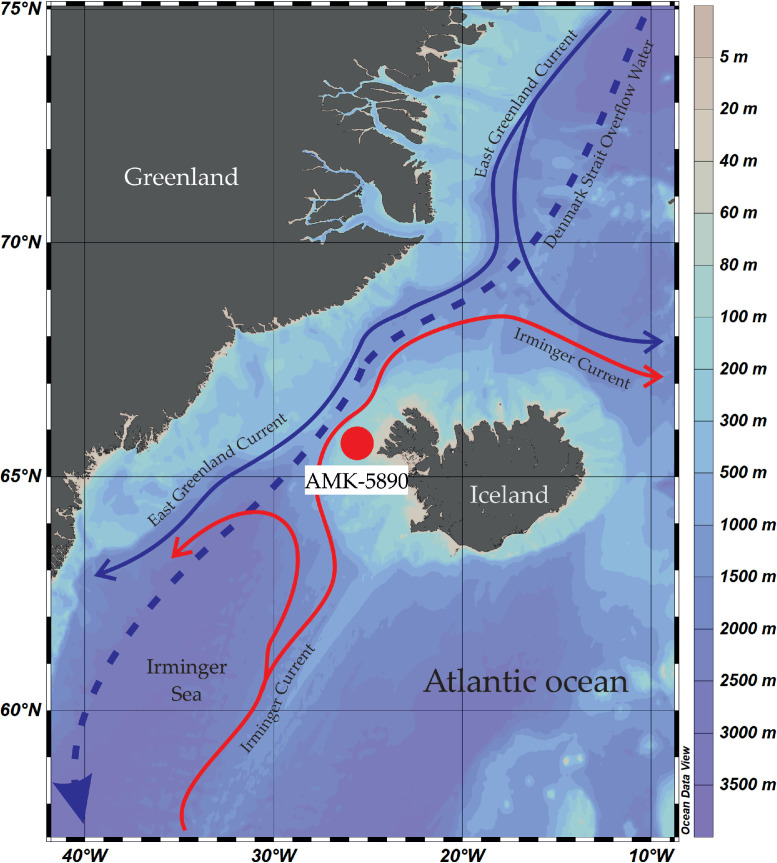
Location map of station AMK-5890 in Denmark Strait on northwest slope Iceland in North Atlantic. Red arrows indicate warm surface current, blue arrows indicate cold surface (solid arrow) and deep (dashed arrow) currents [9,10].

We present:

- map of the northwestern North Atlantic Ocean showing location of studied sediment core AMK-5890, and modern surface and deep currents in Denmark Strait (Fig. 1);
- list of all benthic foraminifera taxa revealed in sediment samples of the core AMK-5890 (Table 1);
- 6 tables with microphotographs of benthic foraminifera taxa and taxonomic descriptions to them (Phototables 1 to 6).

## 2 Experimental design, materials and methods

During the 71st cruise of the R/V *Akademic Mstislav Keldysh* in the summer 2018, the gravity core at the station AMK-5890 retrieved 4.24 m long sediment sequence on the northwestern slope of Iceland in Denmark Strait. Sediments are presented by alternation of light-brown sandy clay with the predominance of carbonate biogenic material, grey sandy-silty-clay, and brown silty-clay, sometimes with gravel inclusions [1]. We analyzed 148 samples (thickness of 1 cm) with spacing of every 1 (core intervals of 10–65 cm and 155–180 cm) to 5 cm of the core. All samples were dried, weighted, and washed through a sieve with mesh size of 63 to save the small-sized foraminiferal tests [2]. Then, as recommended by Fatela and Taborda [3], 150–300 specimens of benthic foraminifera were counted in each sample fraction of >63 µm under the Nikon microscope SMZ800N with a magnification of 80x. To identify benthic foraminifera species, we used publications of Jones [4], Feyling-Hanssen et al. [5], Holbourn et al [6]., Saidova [7], Tikhonova et al [8]. Benthic foraminifera tests were photographed on the Nikon microscope SMZ25 equipped with a Nikon camera DS-Fi3 and NIS-Elements D software. Thereafter, we made phototables using the computer softwares Adobe Photoshop CC 2019 and CorelDRAW 2020.

**Table 1:** List of benthic foraminiferal taxa.

Benthic foraminiferal genera and species
*Alabaminella weddellensis* (Earland, 1936)
*Astrononion gallowayi* Loeblich &Tappan, 1953 = *Astrononion hamadaense* Asano, 1950
*Buccella frigida* (Cushman, 1921) = *Buccella calida* (Cushman & Cole, 1930)
*Buccella tenerrima* (Bandy, 1950) *Bolivina pseudoplicata* Heron-Allen & Earland, 1930
*Bolivina* sp. d'Orbigny, 1839
*Brizalina* sp. Costa, 1856
Bulimina aculeata d'Orbigny, 1826
*Bulimina marginata* d'Orbigny, 1826
*Cassidulina carinata* Silvestri, 1896
*Cassidulina laevigata* d'Orbigny, 1826 *Cassidulina obtusa* Williamson, 1858
*Cassidulina reniforme* Nørvang, 1945
*Cassidulina teretis* Tappan, 1951
*Cassidulinoides bradyi* (Norman, 1881) = *Evolvocassidulina bradyi* (Norman, 1881)
*Cibicides lobatulus* (Walker &Jacob, 1798)
*Cibicides refulgens* Montfort, 1808
*Cibicidoides pseudoungerianus* (Cushman, 1922)
*Cibicidoides wuellerstorfi* (Schwager, 1866)
*Cibicidoides* sp*.*Thalmann, 1939
*Dentalina* sp. Risso, 1826
*Discorbis* sp. Barker, 1960
*Elphidium albiumbilicatum* (Weiss, 1954)
*Elphidium bartletti* Cushman, 1933
*Elphidium excavatum subsp. clavatum* Cushman, 1930
*Elphidium subarcticum* Cushman, 1944
*Epistominella exigua* (Brady, 1884)
*Fissurina* sp. Reuss, 1850
*Fursenkoina complanata* (Egger, 1893) = *Stainforthia loeblichi* (Feyling-Hanssen, 1954)
*Fursenkoina fusiformis* (Williamson, 1858) = *Stainforthia fusiformis* (Williamson, 1858)
*Glandulina laevigata* (d'Orbigny, 1826)
*Globobulimina pacifica* Cushman, 1927
*Globocassidulina subglobosa* (Brady, 1881)
*Gyroidina orbicularis* d'Orbigny in Parker, Jones &Brady, 1865
*Haynesina orbicularis* (Brady, 1881)
*Hyalinea balthica* (Schr€oter in Gmelin, 1791)
*Islandiella helenae* Feyling-Hanssen &Buzas, 1976
*Islandiella norcrossi* (Cushman, 1933)
*Lagena* sp. Walker & Jacob, 1798
*Lenticulina gibba* (d'Orbigny, 1839)
*Melonis barleeanus* (Williamson, 1858)
*Melonis pompilioides* (Fichtel &Moll, 1798)
*Nonion labradoricum* (Dawson, 1860)
*Nonion umbilicatulum* (Walker & Jacob, 1798)
*Nonionella auricula* Heron-Allen &Earland, 1930
*Nonionella turgida* (Williamson, 1858) = *Nonionoides turgidus* (Williamson, 1858)
*Oolina* sp. d'Orbigny, 1839
*Oridorsalis umbonatus* (Reuss, 1851)
*Planulina* sp. d'Orbigny, 1826
*Pullenia bulloides* (d'Orbigny, 1846)
*Pullenia quinqueloba* (Reuss, 1851)
*Pullenia* sp. Parker &Jones, 1862
*Pyrgo* sp. Defrance, 1824
*Quinqueloculina seminula* (Linnaeus, 1758)
*Robertinoides bradyi* (Cushman & Parker, 1936)
*Rosalina villardeboana* d'Orbigny, 1839 = *Discorbis vilardeboanus* (d'Orbigny, 1839)
*Stainforthia concava* (Höglund, 1947)
*Trifarina angulosa* (Williamson, 1858)
*Trifarina fluens* (Todd in Cushman &McCulloch, 1948)
*Trifarina* sp. Cushman, 1923 *Triloculina trihedra* Loeblich & Tappan, 1953
*Uvigerina mediterranea* Hofker, 1932
*Uvigerina peregrina* Cushman, 1923

**Phototable 1:**
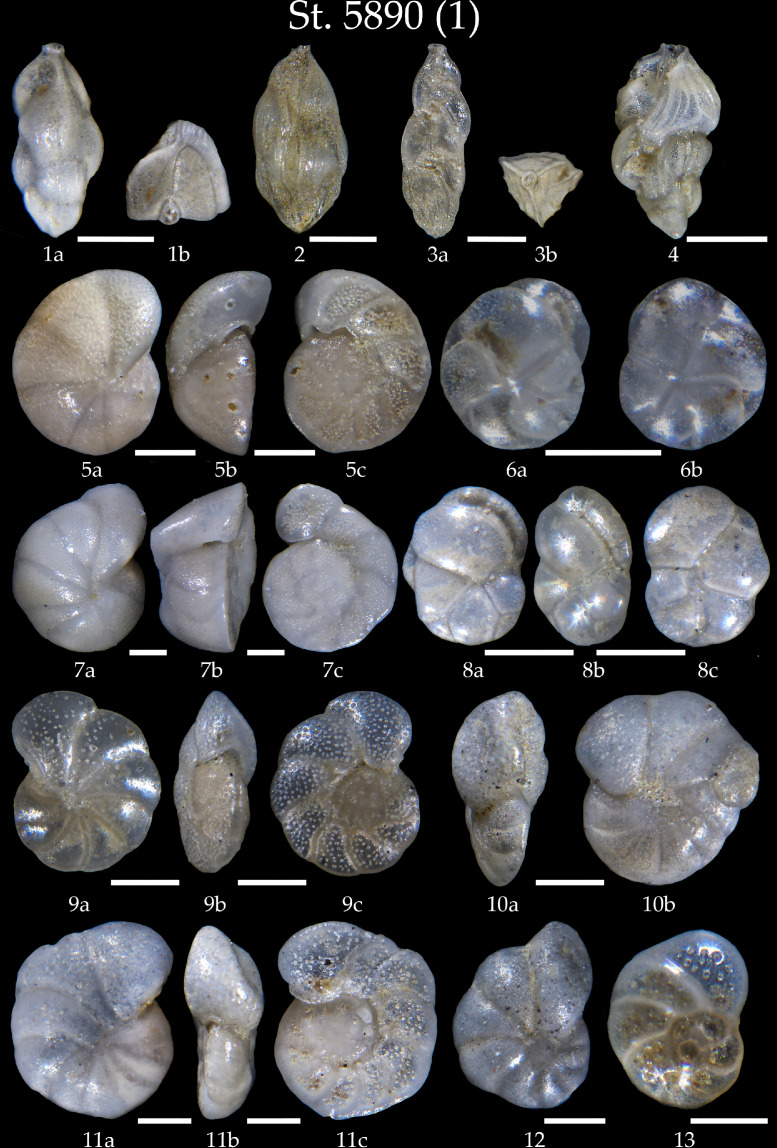
Station 5890. **1, 2** *Trifarina angulosa*, **1a** side view, **2b** apertural view. **3, 4** *Trifarina fluens*, **3a** side view, **3b** apertural view. **5** *Cibicides lobatulus*, **a** spiral view, **b** apertural view, **c** umbilical view. **6** *Cassidulina reniforme*, **a** apertural view, **b** lateral view. **7** *Cibicides refulgens*, **a** spiral view, **b** apertural view, **c** umbilical view. **8** *Cassidulina obtuse*, **a, b** apertural view, **c** lateral view. **9** *Cibicidoides wuellerstorfi*, **a** spiral view, **b** apertural view, **c** umbilical view. **10** *Cibicidoides* sp., **a** apertural view, **b** spiral view. **11** *Cibicidoides pseudoungerianus*, **a** spiral view, **b** apertural view, **c** umbilical view. **12** *Cibicidoides* sp. **13** *Planulina* sp. Scale 100 µm.

**Phototable 2:**
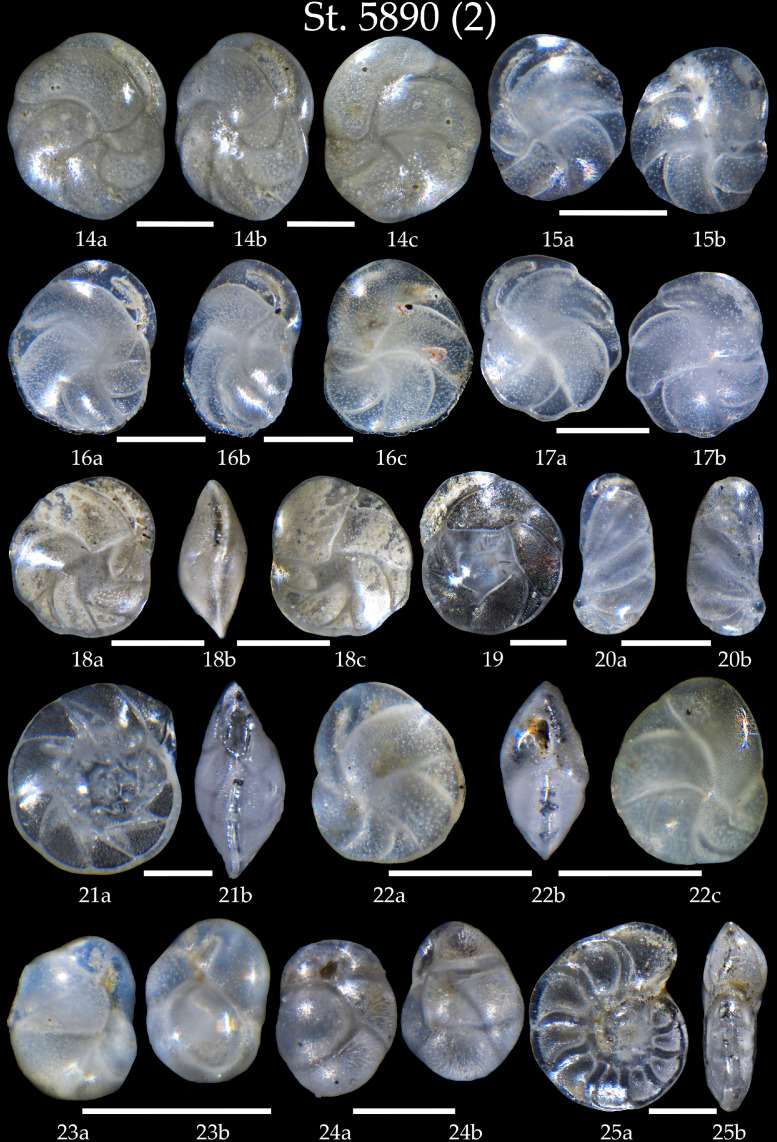
Station 5890 *(continued)*. **14** *Cassidulina laevigata*, **a, b** apertural view, **c** lateral view. **15** *C. laevigata*, **a** apertural view, **b** lateral view. **16** *Cassidulina carinata*, **a, b** apertural view, **c** lateral view. **17** *C. carinata*, **a** apertural view, **b** lateral view. **18** *Cassidulina teretis*, **a, b** apertural view, **c** lateral view. **19** *C. teretis*. **20** *Cassidulinoides bradyi*, **a** apertural view, **b** lateral view. **21** *Islandiella helenae*, **a** apertural view, **b** lateral view. **22** *Islandiella norcrossi* **a, b** apertural view, **c** lateral view. **23, 24** *Globocassidulina subglobosa*, **23a** side view, **23b** apertural view, **24a** apertural view, **24b** side view. **25** *Hyalinea balthica*, **a** side view, b apertural view. Scale 100 µm.

**Phototable 3:**
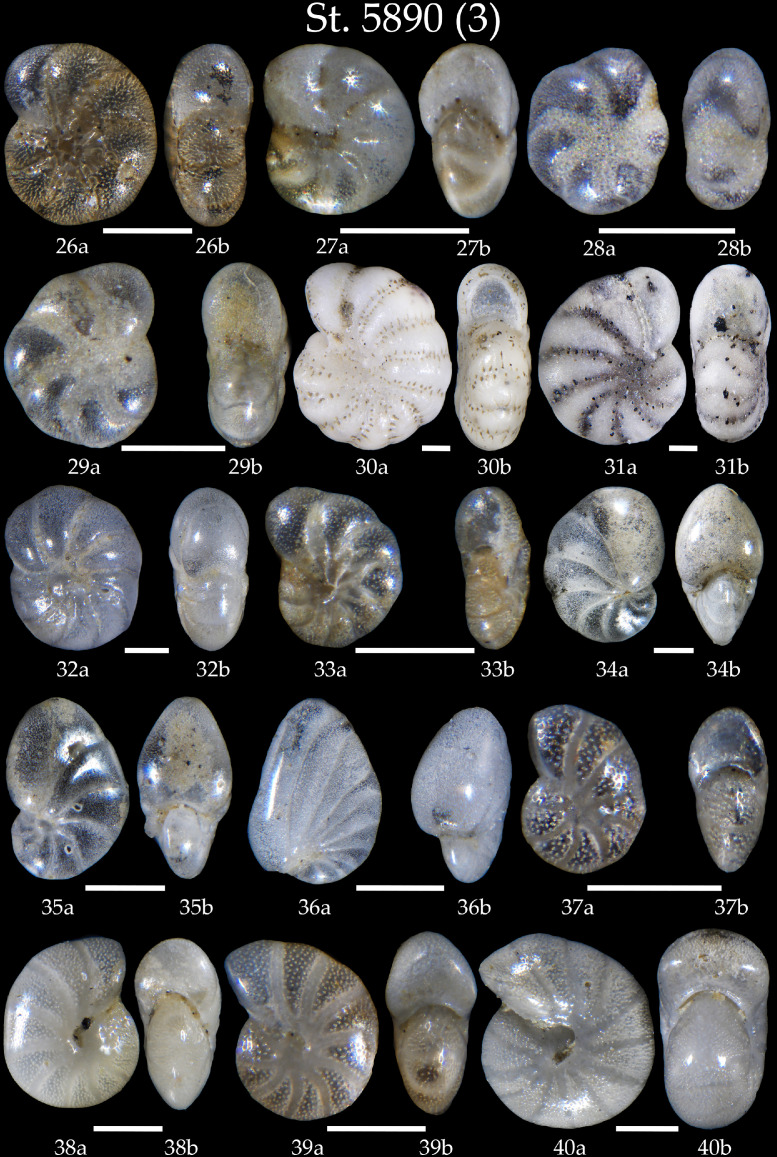
Station 5890 *(continued)*. **26, 27** *Elphidium excavatum* subsp. *clavatum*, **a** side view, **b** apertural view. **28** *Elphidium albiumbilicatum*, **a** side view, **b** apertural view. **29** *Elphidium subarcticum*, **a** side view, **b** apertural view. **30, 31** *Elphidium bartletti*, **a** side view, **b** apertural view. **32** *Haynesina orbicularis*, **a** side view, **b** apertural view. **33** *Astrononion gallowayi*, **a** side view, **b** apertural view. **34** *Nonion labradoricum*, **a** side view, **b** apertural view. **35** *Nonionella auricula*, **a** side view, **b** apertural view. **36** *Nonionella turgida*, **a** side view, **b** apertural view. **37** *Nonion umbilicatulum*, **a** side view, **b** apertural view. **38, 39** *Melonis barleeanus*, **a** side view, **b** apertural view. **40** *Melonis pompilioides*, **a** side view, **b** apertural view. Scale 100 µm.

**Phototable 4:**
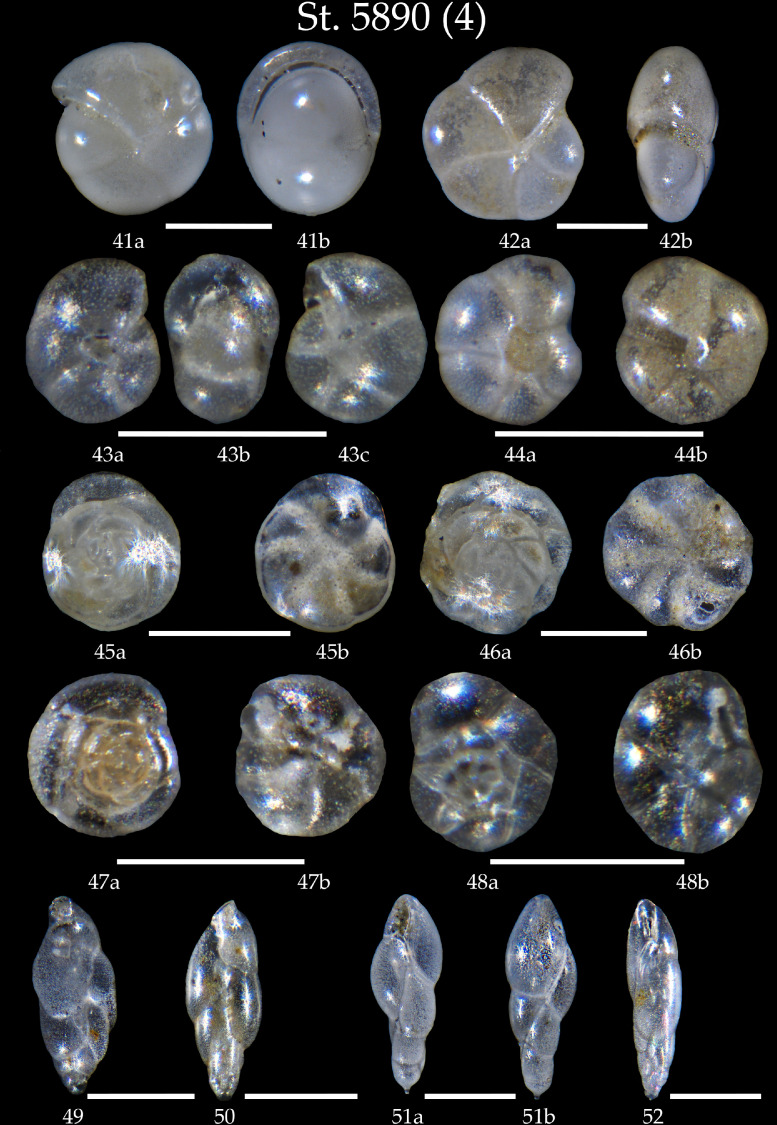
Station 5890 (continued). **41** *Pullenia bulloides*, **a** side view, **b** apertural view. **42** *Pullenia quinqueloba*, **a** side view, **b** apertural view. **43, 44** *Pullenia* sp., **43a** umbilical view, **43b** apertural view, **43c** lateral view, **44a** umbilical view, **44b** lateral view. **45** *Buccella frigida*, **a** spiral view, **b** umbilical view. **46** *Buccella tenerrima*, **a** spiral view, **b** umbilical view. **47** *Alabaminella weddellensis*, **a** spiral view, **b** umbilical view. **48** *Epistominella exigua*, **a** spiral view, **b** umbilical view. **49, 50** *Fursenkoina fusiformis*. **51, 52** *Fursenkoina complanata*, **51a** apertural view, **51b** side view. Scale 100 µm.

**Phototable 5:**
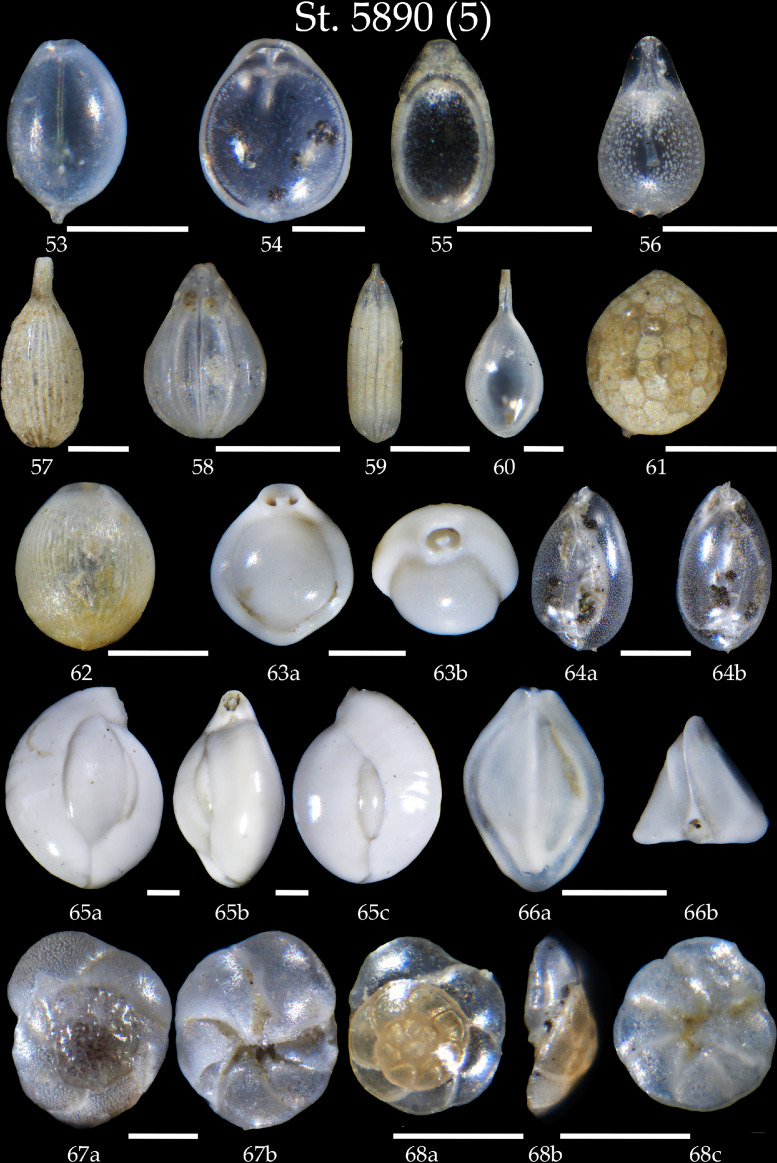
Station 5890 (continued). **53-56** *Fissurina* sp. **57-60** *Lagena* sp. **61, 62** *Oolina* sp. **63** *Pyrgo* sp. cf. *P. murrhina*. **64** *Globobulimina pacifica*, **a** side view, **b** apertural view. **65** *Quinqueloculina seminula* **a** side view **b** apertural view, **c** lateral view. **66** *Triloculina trihedra* **a** lateral view, **b** apertural view. **67** *Discorbis* sp., **a** spiral view, **b** umbilical view. **68** *Rosalina villardeboana*, **a** spiral view, **b** apertural view, **c** umbilical view. Scale 100 µm.

**Phototable 6:**
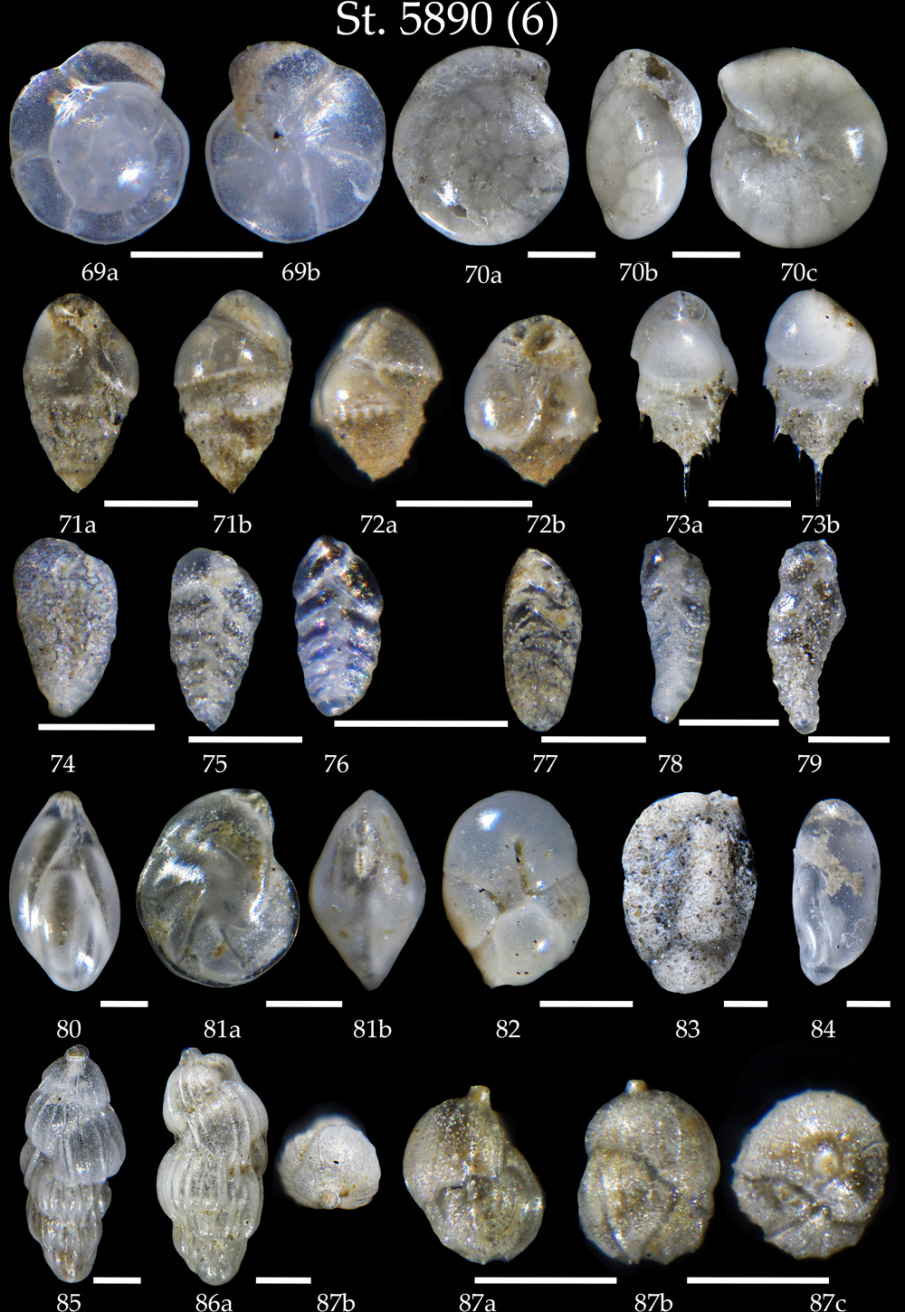
Station 5890 (continued). **69** *Oridorsalis umbonatus*, **a** spiral view, **b** umbilical view. **70** *Gyroidina orbicularis*, **a** spiral view, **b** apertural view, **c** umbilical view. **71, 72** *Bulimina marginata*, **71a** apertural view, **71b** side view, **72a** side view, **72b** apertural view. **73** *Bulimina aculeata*, **a** apertural view, **b** side view. **74** *Bolivina pseudoplicata*. **75-76** *Bolivina* sp. **77-79** *Brizalina* sp. **80** *Glandulina laevigata*. **81** *Lenticulina gibba*, **a** apertural view, **b** umbilical view. **82** *Robertinoides bradyi*. **83** ? **84** ? **85, 86** *Uvigerina peregrina*, **86a** side view, **86b** apertural view. **87** *Uvigerina mediterranea*, **a,b** side view, **c** apertural view. Scale 100 µm.

## CRediT authorship contribution statement

**Liubov Kireenko:** Conceptualization, Investigation, Methodology, Formal analysis, Resources, Writing – original draft, Visualization, Funding acquisition. **Anna Tikhonova:** Investigation, Writing – review & editing, Resources. **Nina Kozina:** Writing – review & editing, Resources. **Alexander Matul:** Funding acquisition, Writing – review & editing.

## Declaration of Competing Interest

The authors declare that they have no known competing financial interests or personal relationships that could have appeared to influence the work reported in this paper.
